# Deep multi-modal learning for joint linear representation of nonlinear dynamical systems

**DOI:** 10.1038/s41598-022-15669-7

**Published:** 2022-07-27

**Authors:** Shaodi Qian, Chun-An Chou, Jr-Shin Li

**Affiliations:** 1 Mechanical and Industrial Engineering, Northeastern University, Boston, MA 02215 USA; 2 Electrical and Systems Engineering, Washington University in St. Louis, St. Louis, MO 63130 USA

**Keywords:** Neuroscience, Biomarkers, Engineering

## Abstract

Dynamical systems pervasively seen in most real-life applications are complex and behave by following certain evolution rules or dynamical patterns, which are linear, non-linear, or stochastic. The underlying dynamics (or evolution rule) of such complex systems, if found, can be used for understanding the system behavior, and furthermore for system prediction and control. It is common to analyze the system’s dynamics through observations in different modality approaches. For instance, to recognize patient deterioration in acute care, it usually relies on monitoring and analyzing vital signs and other observations, such as blood pressure, heart rate, respiration, and electroencephalography. These observations convey the information describing the same target system, but the dynamics is not able to be directly characterized due to high complexity of individual modality and maybe time-delay interactions among modalities. In this work, we suppose that the state behavior of a dynamical system follows an intrinsic dynamics shared among these modalities. We specifically propose a new deep auto-encoder framework using the Koopman operator theory to derive the joint linear dynamics for a target system in a space spanned by the intrinsic coordinates. The proposed method aims to reconstruct the original system states by learning the information provided among multiple modalities. Furthermore, with the derived intrinsic dynamics, our method is capable of restoring the missing observations within and across modalities, and used for predicting the future states of the system that follows the same evolution rule.

## Introduction

**Figure 1:**
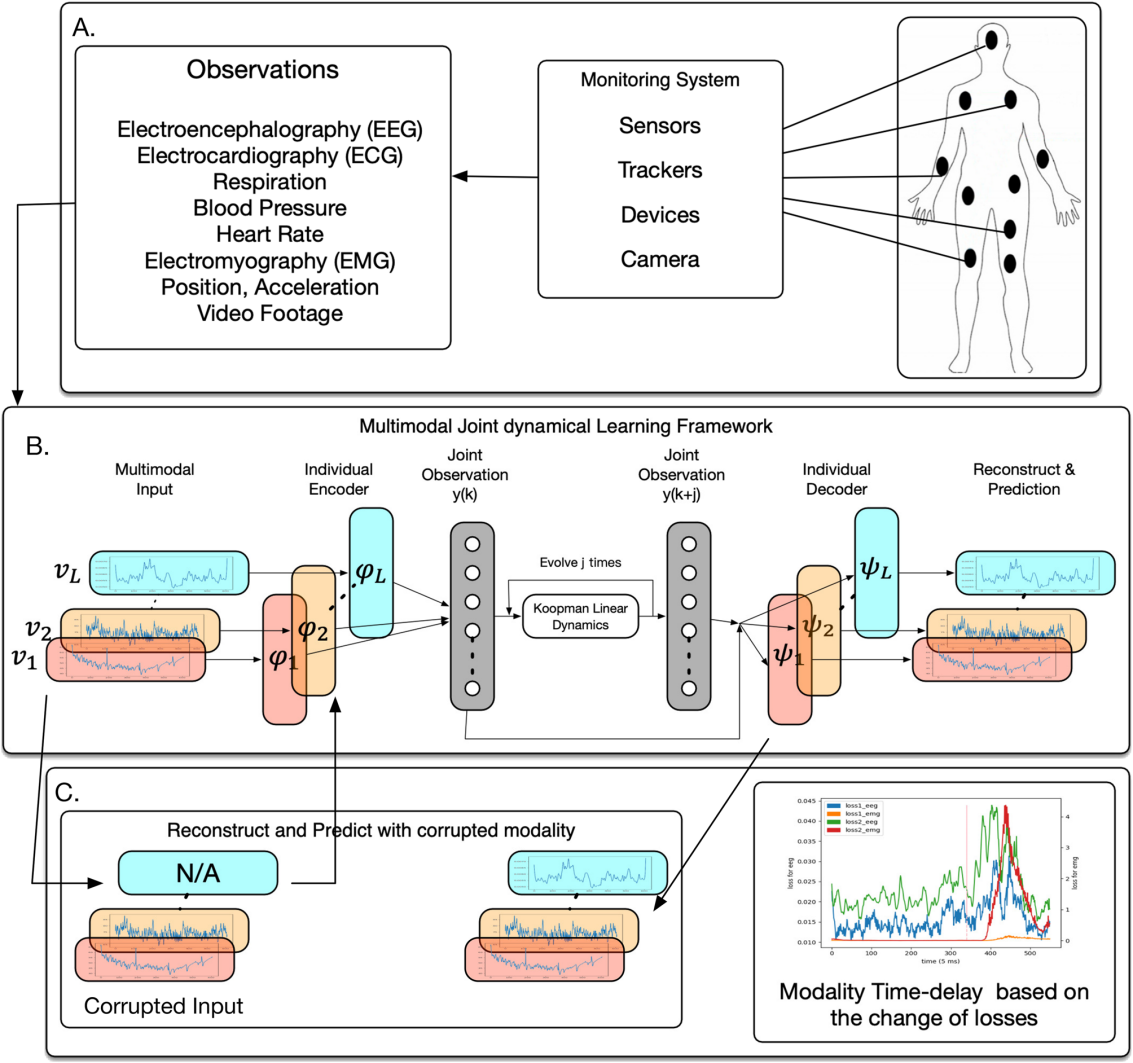
An illustration of the proposed Koopman-operator based multi-modal deep auto-encoder network for the joint intrinsic dynamics of a dynamical system.

Dynamical systems are pervasively seen in a wide range of real-life applications such as neuroscience, healthcare, biology, and engineering^1–4^. These systems are oftentimes complex and behave by following certain evolution rules or dynamical patterns (called dynamics), which are linear, non-linear, or stochastic. Discovering the evolution rule of a target system can be beneficial for understanding the system’s behavior, and furthermore for prediction and control of the future system state.

It is common to analyze the system’s dynamics through observations in different modality approaches. Different modalities from different sources in different dimensions are used depending on systems to be studied. For example of emotion recognition, emotional states are recognized/differentiated based on simultaneously physiological reactions (such as brain’s electrical activity, heart’s electrical activity, muscle’s electrical activity, etc.), facial expression, and/or gestures of the human body system^5^. These observations generally are defined as *multi-modal data* in data analysis and modeling, and convey specific information to represent the system’s behaviors or dynamics. These modalities have somehow correlations with each other as they measure the same dynamical system from different perspectives. It is reasonable to hypothesize that they share certain information of the system. However, the shared information may be very limited and not directly discernible. In addition, there may be time-delay correlations existing among different modalities. Lastly, the quality of multi-modal and/or multi-variate data suffer from noisy background and missing/corrupted observations. Therefore, finding the underlying dynamics from these high-dimensional multi-modal data is not straightforward and remains a challenging task in data analysis of dynamical system. In this study, we propose a deep auto-encoder network to discover the shared dynamics of the dynamical system from multi-modal observations based on dynamical systems theory. Moreover, our method is designed to reconstruct and predict the trajectories of different modalities based on the shared intrinsic dynamics even when part of modalities are corrupted or contaminated with noises.

In general, multi-modal learning and prediction models can be categorized into two main classes: feature-level fusion and data-level fusion. For feature-level fusion, it is popular to extract features from multiple modalities separately and further concatenate uni-modality features into a single feature set. For each modality, state-of-the-art feature engineering methods, such as time-frequency based, nonlinear, spectral, wavelet, and decomposition techniques can be applied to extract features^6^. The size of this fused feature set increases dramatically with the number of modality dimensions. This typically causes an ill-posed problem in machine learning. Then, it is suggested to implement a separate selection task to select important features and form a refined feature set as the input to machine learning algorithms, including support vector machine (SVM)^7^, decision tree^8^, random forest^9^, gradient boosting algorithms^10^ and clustering algorithms^11^. Such feature-level fusion methods rarely take into consideration the joint or shared information across multiple modalities, which make it difficult to discover ‘true’ dynamical representations or patterns. On the contrary, data-level fusion methods focus on extracting the shared information (i.e., dynamics) across multiple modalities at the same time. This shared pattern is expected to extract and reveal the joint information that explains the dynamical patterns of the target system. For example, Suk proposed a modified multi-modal Deep Boltzmann Machine to discover the complex shared information inherent in both magnetic resonance imaging (MRI) and positron emission tomography (PET) to identify the subjects with Alzheimer’s Disease^12^. The result showed that their data-level fusion deep learning model can learn high-level latent features across multiple modalities, and outperform other state-of-the-art feature-level fusion methods. However, the latent patterns extracted from above-mentioned methods do not show a connection to certain evolution rules. It is difficult to project/predict the future system states based on these latent patterns.

To make predictions of the future system state, directly modeling the target dynamical system by the means of discovering its joint intrinsic dynamics from all kinds of observations is preferable. However, such intrinsic dynamics are very complex and nonlinear, making them impossible to be analyzed directly. From the perspective of dynamic systems theory, local or global linearization is a common way to model and simplify nonlinear dynamics. The Koopman operator theory provides a linear but infinite-dimensional operator to globally linearize the nonlinear dynamics^13^. Since it is hard to represent an infinite-dimensional operator, a finite approximation is necessary for modeling and calculations. Then, the nonlinear dynamics can be represented by a linear model in the Koopman intrinsic space. A mathematical example of a simple linear time-invariant model is $x(k+1)=Ax(k)$, where *x*(*k*) is the system state at discrete time *k* and *A* is the linear approximation of the nonlinear dynamics. Dynamic mode decomposition (DMD) is a popular tool to find the finite approximation of the Koopman intrinsic space^14–16^. The new observables are projected onto proper orthogonal decomposition modes (POD) as singular value decomposition (SVD); however, the performance can be improved by introducing a proper dictionary as a projection basis, which is proposed as extended dynamic mode decomposition (EDMD)^17–19^. Further, researchers study the connections between mode decomposition and tensor component analysis^20^. These methods investigated decomposition methods to obtain the intrinsic space and the observations/variables in such a space; however, the project/mapping functions can be highly complex and nonlinear. While deep learning has demonstrated its capacity to fit complex functions, auto-encoder-based frameworks could be modified to learn the observation functions. Lusch^21^ proposed a framework to use an auto-encoder with an auxiliary network to discover the representations of Koopman functions from nonlinear simulation data. Similarly, Morton^22^ trained an auto-encoder-based network to find probability observations and probabilistic dynamics instead of regular observations and dynamics. However, the above methods are designed for uni-variate time series cases. In a multi-modal case, an invariant pattern without dynamics is widely studied, i.e., spatial pattern or compressed information. For example, individual auto-encoder can be constructed to deal with different data sources^23^. Jaques^24^ developed a multi-modal auto-encoder model to extract features from different data sources, including surveys, physiology signals, location, weather, SMS, and so on to predict the stress level. This model can restore missing modalities based on the patterns learnt from training data. However, this study did not focus on finding shared/joint dynamical patterns; instead, they concatenated hidden layers into a feature vector to predict the stress level. Du^25^ developed a multi-modal auto-encoder with a polynomial fusion layer to obtain the joint pattern by introducing a polynomial fusion layer. However, a common problem for deep learning networks is low interpretability, where the variables in the hidden layer do not have physical meanings. To discover joint dynamics from multi-modal time series, in this study, we develop a multi-modal deep learning network to discover the joint dynamics, which can explain the evolution of all modalities simultaneously. The nodes in the joint hidden layer, which fuse the information from all modalities, are the observations in the Koopman linear space. The evolution rule for these variables has the same dynamical properties as the Koopman operator.

It is worth mentioning several challenges to be considered when finding a joint linear dynamics to explain the evolution of all modalities: (1) different modalities may have opposite dynamical behaviors although sharing the same underlying dynamics; (2) time-delay may exist across different modalities; and (3) the joint dynamics should have physical properties that can be recognized as a linear dynamical system. Most prior studies have attempted to address one of these issues; however, it is difficult to overcome all difficulties with a single model.

In this study, we assume that a joint dynamics exists across modalities used to monitor and measure the same dynamical system. Our framework, as illustrated in Fig. 1 will learn a shared dynamical system to reconstruct and predict the dynamics of all modalities. Even if parts of the modalities are missing, the missing modalities can be restored and predicted based on other well-preserved modalities and the intrinsic dynamical model. After obtaining the intrinsic dynamical model, tools from linear dynamical systems can be applied to the hidden layer. To validate our method, we consider real-life applications, where human body system is modeled as a dynamical system of multiple physiological modalities. We aim to assess and further predict the human physiological reactions under different driving stimuli in a driving simulation environment^26^.

## Results

### Koopman operator theory

First, let us consider a dynamical system as:

1 $$\begin{aligned} \frac{d}{dt}y(t) = h(y(t), t), \end{aligned}$$

where *h* is the dynamics for a target system, and *y*(*t*) is the system state at time *t*. However, in most real-life cases, *h* and *y* can not be obtained or observed directly. In our study, we analyze the target system in a relatively short time window, therefore, we assume that the intrinsic dynamics *h* does not change in this short time window, which means *h* can be treated as an autonomous system. We then simplify the dynamical system as:

2 $$\begin{aligned} \frac{d}{dt}y(t) = h(y(t)), \end{aligned}$$

The system is usually analyzed by observations $x_l$ from different sources, which can be represented by:

3 $$\begin{aligned} x_l(t)={\mathbf {g}}_l(y(t)), \end{aligned}$$

where ${\mathbf {g}}_l$ is the observation function corresponding to modality *l*, and $x_l(t)$ is the system state of the modality *l* at time t. Instead of analyzing the observations $x_l$ separately as most standard studies, our model is designed to uncover the intrinsic dynamics *h*, which can reconstruct and predict the state of all modalities. The reconstruction and prediction for the missing modalities are not possible for most of the previous studies, since they analyze the modalities separately. In our model, we utilize the Koopman operator theory to capture the evolution rule of the system.

The basic idea of the Koopman operator ${\mathscr {K}}$ is to use a finite-dimensional matrix *A* to approximate the evolution of the nonlinear dynamics *h* from all modalities. First, consider a dynamical system for multiple modalities ($l\in L$) as follows:

4 $$\begin{aligned} \frac{d}{dt}x_l(t) = f_l(x_l(t)), \end{aligned}$$

where $x_l(t)$ is the state of modality *l* at time *t*, $f_l$ is the corresponding dynamics. The data are often collected in discrete time *k* as follows:

5 $$\begin{aligned} x_l(k+1) = F_l(x_l(k))=x_l(k)+\int _{k}^{k+\Delta t}f_l(x_l(\tau ))d\tau . \end{aligned}$$

Based on the Koopman theory, we can find a measurement function $\hat{g}_l$ for modality *l* in a function space $\hat{g}$ satisfying Eq. (6):

6 $$\begin{aligned} {\mathscr {K}}\hat{g}_l=\hat{g}_l\circ F_l, \end{aligned}$$

where $\circ$ is the composition operator. In this representation, we can write the evolution of the system state *x* at time point *k* as:

7 $$\begin{aligned} {\mathscr {K}}\hat{g}_l(x_k)=\hat{g}_l(F_l(x_k))=\hat{g}_l(x_{k+1}). \end{aligned}$$

This function $\hat{g}_l$ can be treated as an inverse-observation function for modality *l*, which projects the observation/modality *l* back to the same intrinsic variables $\hat{y}$ separately. We further simplify the linear dynamics as follows $\hat{y}(k+1)=A\hat{y}(k)$, where $\hat{y}(k)$ is the approximation of system state at discrete time *k* and *A* is the linear approximation of the Koopman operator. The future system states will be predicted based on the initial states and the learned linear dynamics.

### Multi-modal deep learning via Koopman operator

The observation functions for different modalities can be highly complex and nonlinear, which makes it hard to uncover the shared information. Since deep learning is a powerful tool to fit complex nonlinear functions, we designed a modified multi-modal auto-encoder to uncover the shared dynamics from multiple modalities. auto-encoder is a neural network consisting of two parts: an encoder which maps the input into the hidden representation and a decoder which maps the hidden representation back to a reconstructed input. For each modality $l \in L$, an encoder will learn a mapping function, inverse-observation function, $\varphi _l$ from the input variables to a shared hidden layer; at the same time, a decoder will learn to reconstruct the input variables from the variables in the hidden layer via an approximation of observation function $\varphi _l^{-1}$. The shared layer $\hat{y}(k)$ is defined as: $\hat{y}(k)=\frac{1}{L}\sum _{l\in L(not\ missing)}\hat{y}_l(k)$, where $\hat{y}_l(k)$ is the encoded observation for modality *l* at time *k*. The shared layer contains the variables of a linear dynamics, which is a finite approximation of the Koopman operator, and the dimension of this shared layer is a pre-determined hyper-parameter. In our studies, the dimension of the intrinsic linear dynamics is set to 20. The basic reconstruction error of an auto-encoder can be written as: $L_{recon}=L({\mathbf {x}}_l, {\mathbf {x}}_l') = \Vert {\mathbf {x}}_l - {\mathbf {x}}_l' \Vert ^2$, where ${\mathbf {x}}_l(k)$ and ${\mathbf {x}}_l'(k)$ are the original input vector and reconstructed vector respectively and ${\mathbf {x}}_l'(k) = \varphi _l^{-1}(\varphi _l({\mathbf {x}}_l(k)))$. Through the encoder, the input data is first transformed into the middle embedding layer *Y* . We embed the idea of the Koopman theory into the middle layer to make the variables have linear dynamics in the intrinsic space. To do that, we introduce three more custom loss functions to control the dynamical behavior of the variables in the hidden layer. The first one is prediction loss for prediction states across *m* time points: $L_{predict} = \frac{1}{m} \sum _l \sum _{j=1}^{m} \Vert {\mathbf {x}}_l(k+j) -\varphi _l^{-1}(K^{j} \varphi _l({\mathbf {x}}_l(k))) \Vert$, the second loss is linear dynamics loss defined as: $L_{Linear} = \frac{1}{m} \sum _l \sum _{j=1}^{m} \Vert \varphi _l({\mathbf {x}}_l(k+j))) - K^{j}\varphi _l({\mathbf {x}}_l(k)) \Vert$, the third loss is modality loss defined as: $L_{modality}=\frac{1}{L}\sum _{l\in L(not\ missing)}||y(k)-y_l(k)||^2$, which is similar to the center loss defined in^27^. The prediction loss will measure the difference between the original states and the predictions through the evolution of the hidden linear dynamics. For linear dynamic loss, given a set of input modalities ${\mathbf {x}}$, the auto-encoder will find a corresponding observation ${\mathbf {y}}$ (in the embedding layer) such that the evolution rule for the observation ${\mathbf {y}}$ is linear. Since all modalities are connecting with a shared layer $\hat{y}(k)$ through separate encoders, we construct center loss to minimize the difference between the hidden observations $\hat{y}_l(k)$ across modalities and obtain a center as our joint hidden layer. Since the linear and the original nonlinear dynamics share the same dynamical behavior under the Koopman transformation, the evolution of the input calculated by mapping the hidden variable to the nonlinear space can be reconstructed. By combining these three loss functions, we can constrain the variables in the hidden layer to evolve as linearly as possible. Our model will learn to minimize the loss by combining all three types of losses as follows:

8 $$\begin{aligned} Loss = \lambda _{recon}(L_{recon}+L_{predict})+\lambda _{linear}L_{linear}+\lambda _{modality}L_{modality}+\lambda _{reg} \Vert W \Vert _2^2, \end{aligned}$$

where the last term is a $l_2$ regularization on the weights *W* to avoid a potential overfitting situation and $\lambda _{recon}$ is the penalty term associated with both reconstruction and prediction loss. To be noticed, the scale of different modalities could be different, we can standardize the input data or consider relative loss weights for each modality to improve the performance of the auto-encoder.

### Time delay observation

When the assumption of invariance does not hold or time-delay exists between modalities, which means modalities do not follow the same intrinsic dynamics at time *k*, the previous basic model will have trouble in capturing the joint dynamics. The joint dynamics may converge to a stable state to minimize the loss functions, i.e. the learned pattern is no longer a valid dynamic. Therefore, we rewrite the model to obtain a time-delay observation of the system instead of a regular observation at time *k*. Researchers^28,29^ study the linear dynamics of time-delay embedded system from a Hankel matrix of the signal. Pan^30^ studies the relations between the number of time-delays and the linearity of dynamics. To do that, we can apply time-delay embedding on the time series $x_l=[x_l(0), x_l(1), \dots , x_l(t)]$ as follows:

9 $$\begin{aligned} \hat{x}_l = [x_l(k),x_l(k-\tau ),\dots ,x_l(k-(d-1)\tau )] = \left[ {\begin{array}{*{20}{c}} {x_l(0)} &{} {x_l(1)} &{} \cdots &{} {x_l({k_{m_c}})} \\ \\ {x_l(\tau )} &{} {x_l(\tau +1)} &{} \cdots &{} {x_l({k_{m_c + 1}})} \\ \\ \vdots &{} \vdots &{} \ddots &{} \vdots \\ \\ {x_l((d-1)\tau )} &{} {x_l({k_{m_r + 1}})} &{} \cdots &{} {x_l({k_m})} \\ \end{array}} \right] \end{aligned}$$

replace the input $x_l(k)$ with $[x_l(k),x_l(k-\tau ),\dots ,x_l(k-(d-1)\tau )]$, where *d* and $\tau$ are the dimension and delay setup for time delay embedding. The observation function $y(k)=\varphi (x_l(k))$ can be reformed as:

10 $$\begin{aligned} \hat{y}_l(k) = \varphi _l (x_l(k),x_l(k-\tau ),\dots , x_l(k-(d-1)\tau )) \end{aligned}$$

We add white Gaussian noise with custom signal-to-noise (SNR) to the inputs and randomly mask one modality/variable with a specific value (-1 in our case) to train our model to deal with noisy and missing data in multi-modal and/or multi-variate time series.

### Experimental tests

In this work, to validate the effectiveness of the proposed KMMDL method, we employ two publicly available databases that contain multiple physiological modalities for investigating physiological responses to various driving conditions in the virtual reality environment^31,26^. Our Koopman-based auto-encoder approach is used to capture the overall response pattern of multi-modal physiological system by reconstructing and predicting the joint dynamics.

In the two applications, observations were taken to describe physiological reactions in different circumstances and stimuli. In the first case study, we aim to test the joint linear dynamics extracted from multiple modalities. Theoretically speaking, after the joint dynamical system is obtained, we can reconstruct and predict every modality, even when we only have one available modality. Therefore, we evaluate the reconstruction and prediction accuracy and assess the model performance while one or more modalities are missing. Since our model tends to capture a general dynamical behavior in a certain time range, the error will increase when the dynamics change or unexpected stimuli occur. In the second case study, we used our model to identify the time point when unexpected events occur or dynamics change. We applied our model to capture the joint dynamics from electroencephalogram (EEG) and electromyography (EMG) to predict the upcoming reaction towards stimuli during driving simulation. Through this task, we identify the reaction time from both brain and muscle.

#### Case study 1: responses to distracted conditions

This study aims to study the different physiological patterns under different driving conditions. There are 68 subjects that drove the same highway under eight different conditions: (1) relaxation without driving, (2) practice, (3) free driving on a straight road, (4) driving on straight with surprise unintended acceleration event, (5) normal drive without distraction, (6) cognitive distraction (taking analytical or mathematical questions), (7) emotional distraction (taking emotional questions), and (8) sensorimotor distraction (texting and/or talking). There is a 2-min break between driving tasks. Driving performance, physiological signals, and videos were collected continuously for each driving condition. In particular, physiological signals including palm electrodermal activity (EDA), perinasal perspiration, heart rate, and breathing rate are used in our methodology verification. The original sampling rate for physiological data is 60 Hz and down-sampled to 1 Hz. We want to fuse the information from all physiological signals and try to find whether the physical reaction under different tasks is significantly different. Therefore, we select the subjects with all four loaded driving, including normal drive without distraction, cognitive distraction, emotional distraction, and sensorimotor distraction. Part of the data is corrupted due to invalid data and missing modalities. Therefore, we only keep the sessions with valid data for training. At last, we keep 21 subjects in total. Before feeding into our training model, we add white Gaussian noise with a 10dB signal-to-noise (SNR) ratio.

**Figure 2:**
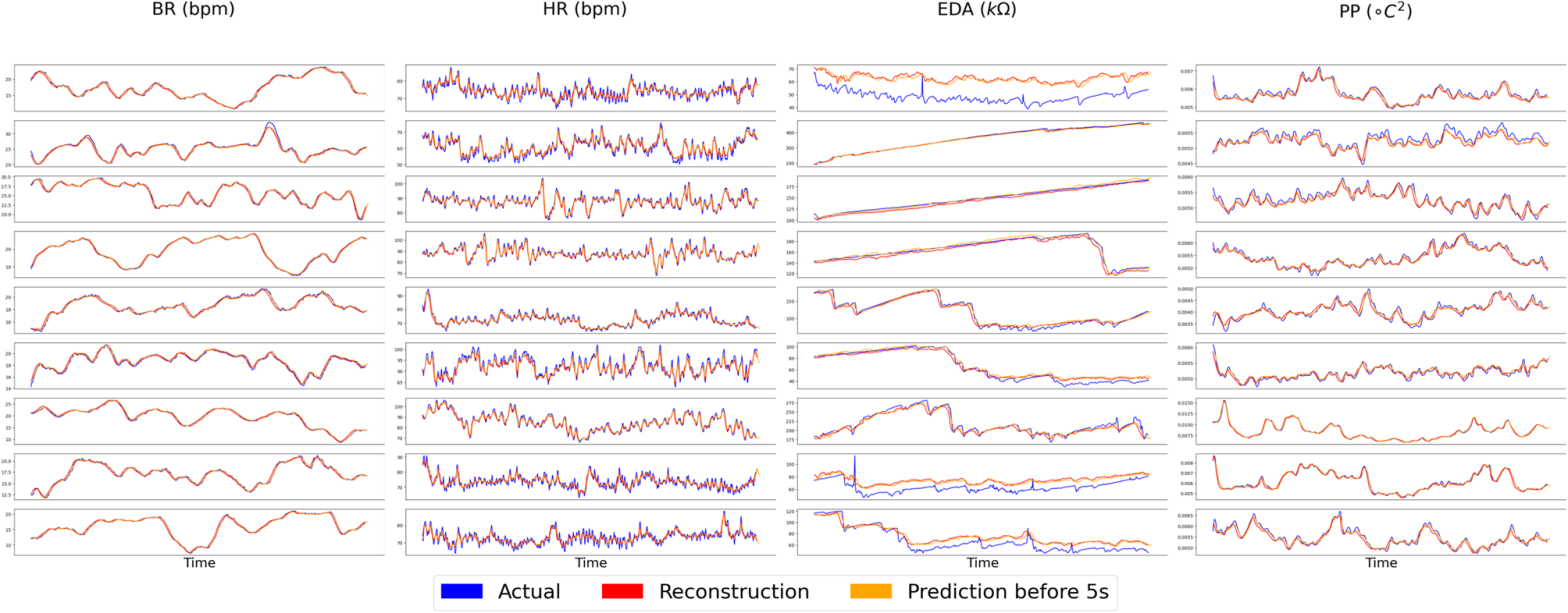
Reconstruction and future prediction made by our model for all nine testing sessions.

From these four modalities, our model extracts a joint dynamics which can explain the changes of all modalities as shown in Fig. 2. The prediction is based on the shared linear dynamics by the system time-delay observation 5 seconds ago. The root mean square errors (RMSE) for reconstruction and prediction are both lower to around $1e^{-2}$. Through this application, our model captures an objective dynamics to explain the intrinsic dynamics for all four modalities from all subjects. Comparing to other modalities, EDA has the worst result in terms of RMSE for reconstruction and prediction. The shared linear dynamics may not be able to predict the future states of the EDA, as the prediction of EDA will tend to converge to stable states. However, the predictions of other modalities are still accurate. This low accuracy of EDA could result from the scale of time-dependency of EDA larger than our time-delay embedding setting.

**Figure 3:**
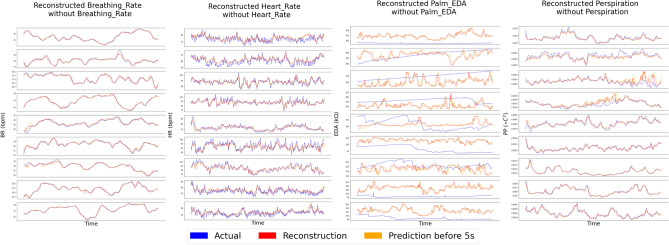
Estimations for missing modalities based on other modalities for all nine testing sessions.

**Figure 4:**
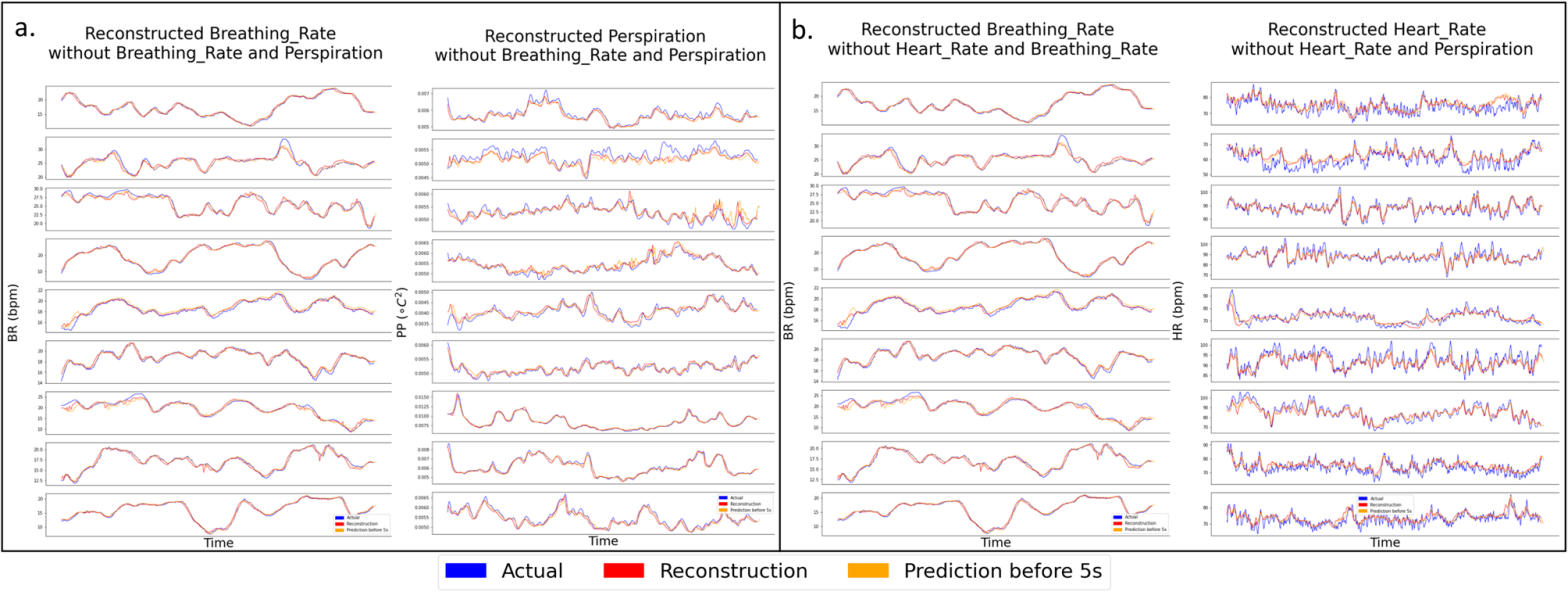
Estimations for missing modalities (2 missing modalities at the same time) based on other modalities for all nine testing sessions. (**a**) Masking breathing rate and perspiration, (**b**) masking heart rate and breathing rate.

Theoretically, after training the model and learning the joint dynamics, one modality can be enough to obtain the information to reconstruct and predict the states of other modalities. In Fig. 3, every modality is masked and restored by other modalities in our framework. To be noted, the modality loss $L_{modality}$ is lower to around $1e^{-4}$ after training. In Fig. 4, two modalities are missing and restored by other available modalities. These results indicate that even if one or more modalities are corrupted with missing values, the missing modalities can be restored or estimated accurately by the other three modalities and the joint linear dynamics is still able to explain the evolution of modalities. The reconstruction and prediction results show the great advantages of our intrinsic model over other models with union information since it is hard and nearly impossible for previous models to reconstruct and predict all possible modalities based on a single model.

#### Case study 2: responses to emergency braking

In this case study, the goal is to detect the upcoming emergency brakings based on real-time EEG and EMG^26^. We extract joint dynamics from high frequency (200 Hz) EEG and EMG data for predicting the upcoming emergency event. Since we expect a spike in the reconstruction and prediction loss when an emergency event happens, we calculate the reaction time based on the change of dynamics after a stimulus (emergence brake) occurs during a car following task. This dataset collected EEG by a 32-electrode-cap and 25 EEG electrodes (F3, Fz, F4, P7, P8, T8, FC3, FC4, C3, Cz, C4, T7, CP3, CP4, FC5, P3, Pz, P4, FC6, O1, O2, Oz, CPz, PO4, PO3) were placed based on the international 10-20 system, and muscle activity was collected by two electrodes placed on the right musculus tibialis anterior and right thigh. Since previous studies^32,33^ indicated that ‘Pz’ EEG channel is a good source to differentiate between sharp braking and no braking event, we trained our model based on EMG and ‘Pz’ channel from EEG. We segmented the EMG and EEG signals from pre-stimulus 340 ms to post-stimulus 240 ms for all stimuli. We trained separate models for each of them, and further analyze their reaction time based on the reconstruction and prediction by our model.

**Figure 5:**
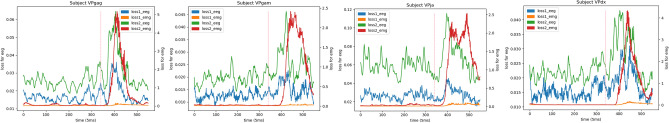
An illustration of reconstruction (loss1) and prediction (loss2) loss corresponding to stimulus/emergency (locate at time 340 ms), which is marked as a pink vertical line.

**Figure 6:**
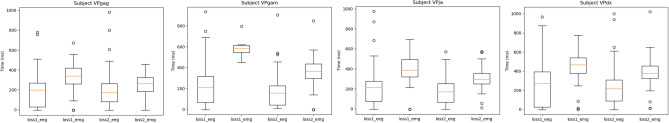
Box plots of reaction time based on both losses from EEG and EMG.

In Fig. 5, we plot the reconstruction and prediction losses through time from four distinct subjects. When the stimulus occurs, it is clear to see that the brain area covered by ‘Pz’ channel reacts faster than muscle. Then, we used the mean value before stimulus as baseline, and recorded the reaction time, which is defined as the first time when all the losses in a given consecutive time length are larger than a ratio of baseline. In our research, we set the required consecutive time length as 3, the ratio for EEG to 1.5, and the ratio for EMG to 4. In Fig. 6, the mean reaction time from ‘Pz’ channel is also faster than muscle activity as suggested in Fig. 5. It is clear that time-delay between EEG and EMG is well preserved by our framework, and reflects the fact that the brain is controlling the movement of muscle.

## Discussion

Figures 2, 3 and 4 indicate that the joint dynamics can reconstruct current system states and predict future system states efficiently even when part of the modalities are corrupted by noise and missing value. Among four different modalities, the performance of EDA is the worst in terms of reconstruction and prediction results, especially when part of the modalities are missing. The potential reasons could be: (1). the variant of EDA is much lower than other modalities, i.e. the EDA does not change much over time; (2). the time-delay of EDA could be larger than the time-delay embedding setup. This makes the linear dynamics fail to present the changes in EDA. This problem can be further studied by using a smaller auto-encoder or setting a larger time-delay parameter. On the contrary, heart rate has a relatively larger variant, therefore, our denoised model will tend to capture a general pattern by ignoring peaks or spikes, which are potentially caused by different kinds of noises. On the other hand, our model gets rid of irrelevant information, and is robust enough to deal with noisy inputs.

Figure 5 demonstrates that when emergency occurs, the brain is the first to respond, followed by the muscles. The reaction time based on EEG as shown in Fig. 6 has more outliers and larger variance than those from EMG, because EEG signal usually contains more noise. However, the reaction is calculated by a naive approach, which could potentially bring errors to the reaction time and the outliers. A sophisticated method can reduce the error brought by uncertainty and improve the reliability of the reaction time. Our current result indicates it is clear that the unexpected event can be reflected by the increasing loss of our model. The reaction time based on the loss is consistent with the results of previous studies.

In summary, we design a powerful multi-modal deep learning network to fuse information from multiple modalities and identify the joint dynamics to explain the evolution of every modality based on the Koopman theory, if such joints dynamics exists. Our framework is designed to linearize multi-modal nonlinear dynamical systems and capture the general dynamical pattern in an intrinsic linear space obtained by a customized multi-modal auto-encoder network if such joint dynamical pattern exists. We add custom loss functions to control the dynamical properties of the nodes in the hidden layer. The framework is optimized to handle corrupted data, including noise and missing value. Our deep learning network can make reconstruction and prediction while maintaining interpretability and physical insight from the perspective of dynamical systems by Koopman operator.

Once a joint linear dynamical system representation is obtained, several directions can be further investigated to utilize the property of linear dynamical system. First, since our mode intend to capture a general invariant dynamics, more work will be required to capture the time-variant dynamics. It may be difficult to pinpoint the cause of the change in dynamics because unexpected changes in dynamics can occur as a result of changes in dynamics or unexpected stimuli. Further, our intrinsic dynamical model can be reformulated by adding control vector and control matrix to control the dynamics and modalities evolving towards desirable system states. After the training the general dynamical pattern is captured, the network can be further connected to a classification framework to determine the states of system through backpropagation. Furthermore, a switched dynamical model can be applied to learn several intrinsic dynamics to simplify different dynamics under different conditions. This multi-modal framework provides a new tool to simplify the system by fusing different kinds of observations (modalities).

## Methods

### Training, validation and testing data

For the distracted driving database in case study 1, the data can be downloaded at https://osf.io/c42cn/files/. The R-Friendly Study Data is preprocessed by R language, the physiological and performance signals are concatenated into a single file and down-sampled at 1 Hz. We only keep the subjects with all four driving tasks including normal driving (ND), Emotional Drive (ED), Cognitive Drive (CD), and Sensorimotor Drive (MD). Then, the database is randomly split into training, validation and testing data sets with a ratio 0.8, 0.1, and 0.1, and all three data sets contain at least one session from four different driving tasks.

In second study, we first segment the original EEG and EMG signals based on the stimulus marker (sampled at 200 Hz). Each segment is a time window from 340 ms pre-stimulus to 240 ms post-stimulus. The data sets is split by time order for each subject: first 70% of data is training data, the next 10% is validation data, and the rest (20%) of data is testing data.

### Deep learning network

For each modality, an auto-encoder is constructed to map from input to shared observations, every auto-encoder contains two fully connected hidden layer, first layer has 20 nodes and second one has 15 nodes and the joint layer contains 20 nodes. Each layer in encoder has the form:

$$\begin{aligned} Y=\varphi (W_ev+b_e), \end{aligned}$$

where $W_e$ and $b_e$ are encoder weight and bias, respectively. In decoding, the formula becomes

$$\begin{aligned} v'=\varphi(W_dY+b_d). \end{aligned}$$

We use the rectified linear unit (ReLU) function as an activation function with the form: $\varphi(x)=max(0,x)$. The linear dynamics or Koopman dynamics is simulated by a fully connected layer without any activation functions with the form: $y(k+1)=Ay(k)$, where the weights of network *A* is the finite approximation of the Koopman operator.

### Parameter setting

Since multiple modalities have different scales, input time series is standardized before time-delay embedding, so that the distribution of every input modality has mean 0 and standard deviation 1.

#### Time-delay embedding

The parameter for embedding, dimension *d* can be estimated by the false nearest-neighbor method (FNN)^34^ and the time delay $\tau$ is estimated as the first local minimum in the mutual information function^35^. For case study 1, dimension *d* is set to 10 and time delay $\tau$ is set to 0. For case study 2, dimension *d* is set to 5 and time delay $\tau$ is set to 2.

#### Penalty terms for loss function

For both cases, penalty terms in Eq. (8), $\lambda _{recon}$, $\lambda _{predict}$, $\lambda _{linear}$, and $\lambda _{modality}$ are set to 1, and $\lambda _{reg}$ for $l_2$ regularization is set to $1e^{-8}$.

#### Number of time to calculating prediction and linear loss

As we discussed in previous section, prediction loss for prediction states across *m* time points in defined as: $L_{predict} = \frac{1}{m} \sum _l \sum _{j=1}^{m} \Vert {\mathbf {x}}_l(k+j) -\varphi _l^{-1}(K^{j} \varphi _l({\mathbf {x}}_l(k))) \Vert$, and linear dynamics loss is defined as: $L_{Linear} = \frac{1}{m} \sum _l \sum _{j=1}^{m} \Vert \varphi _l({\mathbf {x}}_l(k+j))) - K^{j}\varphi _l({\mathbf {x}}_l(k)) \Vert$. For case study 1, *m* is set to 5. For case study 2, *m* is set to 10.

### Training

For both case study, the weights for each layer are initialized by xavier initialization^36^, and the bias is initialized to 0. The models are trained for 7 h on an NVIDIA P100 GPU. The optimizer is Adam optimizer^37^, and learning rate is set to $1e^{-3}$.

## Data Availability

The datasets generated during and/or analysed during the current study are not publicly available since the authors have no ownership of the two databases validated in this research, but precessed data are available from the corresponding author on reasonable request.

## References

[1] Izhikevich, E. M. *Dynamical systems in neuroscience* (MIT press, 2007).

[2] Huang, Y. *et al.* Diagnosis of alzheimer’s disease via multi-modality 3d convolutional neural network. *Frontiers in neuroscience* 509 (2019). 10.3389/fnins.2019.00509 PMC6555226 31213967

[3] Georgatzis, K., Williams, C. & Hawthorne, C. Input-output non-linear dynamical systems applied to physiological condition monitoring. In *Machine Learning for Healthcare Conference*, 1–16 (PMLR, 2016).

[4] Tu, P. N. *Dynamical systems: an introduction with applications in economics and biology* (Springer Science & Business Media, 2012).

[5] Castellano, G., Kessous, L. & Caridakis, G. Emotion recognition through multiple modalities: face, body gesture, speech. In *Affect and emotion in human-computer interaction*, 92–103 (Springer, 2008).

[6] Christ, M., Kempa-Liehr, A. W. & Feindt, M. Distributed and parallel time series feature extraction for industrial big data applications. *arXiv preprint* arXiv:1610.07717 (2016).

[7] Noble WS (2006). What is a support vector machine?. Nature biotechnology.

[8] Safavian SR, Landgrebe D (1991). A survey of decision tree classifier methodology. IEEE transactions on systems, man, and cybernetics.

[9] Belgiu M, Drăguţ L (2016). Random forest in remote sensing: A review of applications and future directions. ISPRS journal of photogrammetry and remote sensing.

[10] Friedman JH (2002). Stochastic gradient boosting. Computational statistics & data analysis.

[11] Xu R, Wunsch DC (2010). Clustering algorithms in biomedical research: a review. IEEE reviews in biomedical engineering.

[12] Suk H-I, Lee S-W, Shen D, Initiative ADN (2014). Hierarchical feature representation and multimodal fusion with deep learning for ad/mci diagnosis. NeuroImage.

[13] Koopman BO (1931). Hamiltonian systems and transformation in hilbert space. Proceedings of the national academy of sciences of the united states of america.

[14] Schmid PJ (2010). Dynamic mode decomposition of numerical and experimental data. Journal of fluid mechanics.

[15] Tu, J. H., Rowley, C. W., Luchtenburg, D. M., Brunton, S. L. & Kutz, J. N. On dynamic mode decomposition: Theory and applications. *arXiv preprint* arXiv:1312.0041 (2013).

[16] Kutz, J. N., Brunton, S. L., Brunton, B. W. & Proctor, J. L. *Dynamic mode decomposition: data-driven modeling of complex systems* (SIAM, 2016).

[17] Williams MO, Kevrekidis IG, Rowley CW (2015). A data-driven approximation of the koopman operator: Extending dynamic mode decomposition. Journal of Nonlinear Science.

[18] Li, Q., Dietrich, F., Bollt, E. M. & Kevrekidis, I. G. Extended dynamic mode decomposition with dictionary learning: A data-driven adaptive spectral decomposition of the koopman operator. *Chaos: An Interdisciplinary Journal of Nonlinear Science* **27**, 103111 (2017). 10.1063/1.4993854 29092410

[19] Korda M, Mezić I (2018). On convergence of extended dynamic mode decomposition to the koopman operator. Journal of Nonlinear Science.

[20] Redman, W. T. On koopman mode decomposition and tensor component analysis. *Chaos: An Interdisciplinary Journal of Nonlinear Science* **31**, 051101 (2021). 10.1063/5.0046325 34240947

[21] Lusch B, Kutz JN, Brunton SL (2018). Deep learning for universal linear embeddings of nonlinear dynamics. Nature communications.

[22] Morton, J., Witherden, F. D. & Kochenderfer, M. J. Deep variational koopman models: Inferring koopman observations for uncertainty-aware dynamics modeling and control. *arXiv preprint* arXiv:1902.09742 (2019).

[23] C. Hong, J. Yu, J. Wan, D. Tao and M. Wang. Multimodal Deep Autoencoder for Human Pose Recovery. *IEEE Transactions on Image Processing* **24**(12), 5659–5670 10.1109/TIP.2015.2487860 (2015). 10.1109/TIP.2015.2487860 26452284

[24] Jaques, N., Taylor, S., Sano, A. & Picard, R. Multimodal autoencoder: A deep learning approach to filling in missing sensor data and enabling better mood prediction. In 2017 Seventh International Conference on Affective Computing and Intelligent Interaction (ACII), 202–208 (IEEE, 2017).

[25] Du, Y., Raman, C., Black, A. W., Morency, L.-P. & Eskenazi, M. Multimodal polynomial fusion for detecting driver distraction. *arXiv preprint* arXiv:1810.10565 (2018).

[26] Haufe S (2014). Electrophysiology-based detection of emergency braking intention in real-world driving. Journal of neural engineering.

[27] Wen, Y., Zhang, K., Li, Z. & Qiao, Y. A discriminative feature learning approach for deep face recognition. In *European conference on computer vision*, 499–515 (Springer, 2016).

[28] Brunton SL, Brunton BW, Proctor JL, Kaiser E, Kutz JN (2017). Chaos as an intermittently forced linear system. Nature communications.

[29] Kamb M, Kaiser E, Brunton SL, Kutz JN (2020). Time-delay observables for koopman: Theory and applications. SIAM Journal on Applied Dynamical Systems.

[30] Pan, S. & Duraisamy, K. On the structure of time-delay embedding in linear models of non-linear dynamical systems. *Chaos: An Interdisciplinary Journal of Nonlinear Science* **30**, 073135 (2020). 10.1063/5.0010886 32752611

[31] Taamneh, S. *et al.* A multimodal dataset for various forms of distracted driving. *Sci Data* **4**(1) 10.1038/sdata.2017.110 (2017). 10.1038/sdata.2017.110 PMC5827115 28809848

[32] Kim, I.-H., Kim, J.-W., Haufe, S. & Lee, S.-W. Detection of braking intention in diverse situations during simulated driving based on eeg feature combination. *Journal of neural engineering* **12**, 016001 (2014). 10.1088/1741-2560/12/1/016001 25426805

[33] Fan, M., Yu, Z., Chou, C.-A., Yen, S.-C. & Lin, Y. Detecting physiological changes in response to sudden events in driving: A nonlinear dynamics approach. In *2020 IEEE/ASME International Conference on Advanced Intelligent Mechatronics (AIM)*, 1537–1542 (IEEE, 2020).

[34] Kennel MB, Brown R, Abarbanel HD (1992). Determining embedding dimension for phase-space reconstruction using a geometrical construction. Physical review A.

[35] Fraser AM, Swinney HL (1986). Independent coordinates for strange attractors from mutual information. Physical review A.

[36] Kumar, S. K. On weight initialization in deep neural networks. *arXiv preprint* arXiv:1704.08863 (2017).

[37] Kingma, D. P. & Ba, J. Adam: A method for stochastic optimization. *arXiv preprint* arXiv:1412.6980 (2014).

